# Library preparation method and DNA source influence endogenous DNA recovery from 100‐year‐old avian museum specimens

**DOI:** 10.1002/ece3.10407

**Published:** 2023-08-09

**Authors:** Amie E. Settlecowski, Ben D. Marks, Joseph D. Manthey

**Affiliations:** ^1^ Bird Collection Gantz Family Collections Center The Field Museum Chicago Illinois USA; ^2^ Department of Biological Sciences Texas Tech University Lubbock Texas USA

**Keywords:** historical DNA, library preparation, museomics, natural history collections, shotgun sequencing

## Abstract

Museum specimens collected prior to cryogenic tissue storage are increasingly being used as genetic resources, and though high‐throughput sequencing is becoming more cost‐efficient, whole genome sequencing (WGS) of historical DNA (hDNA) remains inefficient and costly due to its short fragment sizes and high loads of exogenous DNA, among other factors. It is also unclear how sequencing efficiency is influenced by DNA sources. We aimed to identify the most efficient method and DNA source for collecting WGS data from avian museum specimens. We analyzed low‐coverage WGS from 60 DNA libraries prepared from four American Robin (*Turdus migratorius*) and four Abyssinian Thrush (*Turdus abyssinicus*) specimens collected in the 1920s. We compared DNA source (toepad versus incision‐line skin clip) and three library preparation methods: (1) double‐stranded DNA (dsDNA), single tube (KAPA); (2) single‐stranded DNA (ssDNA), multi‐tube (IDT); and (3) ssDNA, single tube (Claret Bioscience). We found that the ssDNA, multi‐tube method resulted in significantly greater endogenous DNA content, average read length, and sequencing efficiency than the other tested methods. We also tested whether a predigestion step reduced exogenous DNA in libraries from one specimen per species and found promising results that warrant further study. The ~10% increase in average sequencing efficiency of the best‐performing method over a commonly implemented dsDNA library preparation method has the potential to significantly increase WGS coverage of hDNA from bird specimens. Future work should evaluate the threshold for specimen age at which these results hold and how the combination of library preparation method and DNA source influence WGS in other taxa.

## Graphical abstract

We analyzed shallow WGS data from 60 historical DNA libraries collected from ~100‐year‐old museum bird study skins through an experimental design that controlled for variation by individual specimen, age of collection, and taxonomy. We found that of the three library preparation methods that we tested, one single‐stranded DNA method returned significantly higher endogenous DNA content, greater sequencing efficiency, and mean read length than either of the other methods. We also found that when taking into consideration the much larger variance that skin clips have in all the aforementioned metrics, toepads are the better source of historical DNA for WGS from museum bird specimens.

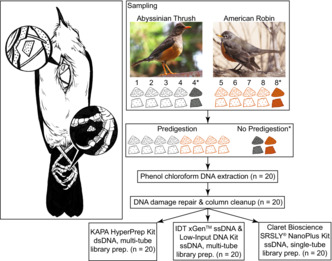

## 1 INTRODUCTION

Museum specimens collected prior to cryogenic tissue storage have long been used as genetic resources to address questions in ecology, evolutionary biology, and conservation (Habel et al., 2014; Wandeler et al., 2007). Genetic studies using these specimens have increased with the advent of high‐throughput sequencing methods, which in comparison to prior Sanger sequencing methods, drastically increase the proportion of degraded DNA that is sequenced (Burrell et al., 2015). Now museum specimens commonly facilitate genomic studies via reduced representation (Bi et al., 2013; Linck et al., 2017; McCormack et al., 2016) and even whole genome sequencing (e.g. van der Valk et al., 2019; Wu et al., 2022). Despite its increasing prevalence and dropping cost, sequencing whole genomes of museum specimens remains expensive because of the degraded nature of the historical DNA (hDNA).

Historical DNA tends to consist of short fragment lengths (McDonough et al., 2018; Straube et al., 2021; Tsai et al., 2020) that are smaller than the recommended library sizes for the most cost‐efficient sequencing setups (i.e., Illumina NovaSeq 6000 S4 flowcell, 200 or 300 cycles). As a result, many sequencing cycles are directly wasted by a lack of base pairs to sequence or indirectly wasted on adapter read‐through (Straube et al., 2021). Historical DNA libraries also tend to consist of low proportions of DNA from the focal specimen (hereafter endogenous DNA). The rest of the library may consist of exogenous DNA from (1) microbes that have colonized the museum specimen or (2) other environmental microbes, (3) contaminating DNA from researchers or (4) other museum specimens, and (5) more recent DNA samples (Fulton & Shapiro, 2019). Altogether, the degraded nature of hDNA results in the recovery of lower proportions of endogenous DNA sequence data (Burrell et al., 2015) and necessitates increased sequencing effort per specimen to recover similar WGS coverage to modern, high‐quality DNA libraries. This inefficiency limits the use of historical DNA from museum specimens to address population genomic questions that require larger sample sizes in addition to sufficient coverage to address questions about selection and demography (Lou et al., 2021).

Ancient DNA researchers have identified that single‐stranded DNA (ssDNA; Bennett et al., 2014; Gansauge & Meyer, 2013; Wales et al., 2015) and single‐tube library preparation methods (Carøe et al., 2018), and those that ligate adapters to unmodified DNA molecule ends (Kapp et al., 2021), increase the amount of degraded ancient DNA molecules that are converted into genomic libraries. However, the most implemented ancient library preparation methods are nonproprietary (Gansauge et al., 2017; Henneberger et al., 2019), thus requiring a high level of startup effort. Early ssDNA methods were also more expensive to implement than double‐stranded DNA (dsDNA) libraries, and their improvement in sequencing efficiency did not warrant the additional effort and cost to implement for all but the most degraded ancient DNA samples (Wales et al., 2015). That is perhaps why only two studies to date have evaluated the influence of ssDNA versus dsDNA library preparation on shotgun sequencing of historical specimens. Sproul and Maddison (2017) found that ssDNA libraries–in comparison to dsDNA libraries–prepared from 16 whole beetle specimens resulted in more retained reads following quality filtering, but no difference in endogenous DNA content. Similarly, Hahn et al. (2022) recently found no difference in endogenous DNA content or insert length between ssDNA and dsDNA libraries prepared from 12 taxonomically diverse wet collection vertebrate specimens. Additional studies of the influence of library preparation on WGS of museum specimens that control for taxonomy, locality, and collection age of specimens will be valuable moving forward. Early ssDNA methods have been modified to reduce costs and ease implementation (Gansauge et al., 2017), and ssDNA methods are now commercially available as kits facilitating further study of their impact on hDNA sequencing efficiency.

Thus far, the majority of research investigating how to maximize the recovery of genetic data from non‐ancient museum specimens has focused on the influence of DNA source or extraction method on DNA yield (Hahn et al., 2022; Hawkins et al., 2022; McDonough et al., 2018; Pacheco et al., 2022; Straube et al., 2021; Tsai et al., 2020; Zacho et al., 2021). However, DNA yield does not necessarily predict sequencing success or efficiency (McDonough et al., 2018; Straube et al., 2021; Zacho et al., 2021) because it is not possible to estimate the proportions of endogenous versus exogenous extracted DNA. For example, a recent study of hundreds of historical genomic DNA libraries built from samples of museum bird specimens found that those built from specimens of smaller species (which generally produce smaller samples) unintuitively had a higher proportion of endogenous sequence data (Irestedt et al., 2022). Moreover, a few studies have shotgun‐sequenced DNA from multiple sources on the same specimen and found differences in endogenous DNA content across sampling sites in fluid‐preserved garter snake specimens (Zacho et al., 2021), prepared mammal skins (McDonough et al., 2018), and formalin‐fixed specimens of a dozen vertebrate taxa (Hahn et al., 2022). Despite research indicating that differences in hDNA sourced from toepads versus incision‐line clips in bird specimens could influence high‐throughput sequencing results (Tsai et al., 2020), no studies have evaluated their difference in endogenous DNA content and sequencing efficiency.

Bird study skin specimens have been an especially prolific source of hDNA research (Billerman & Walsh, 2019) in part due to preservation methods (i.e., skin drying) that are not catastrophic to DNA preservation relative to methods such as formalin‐fixation. Bird study skins have been the foci of some of the earliest studies of hDNA (Mundy et al., 1997), the source for some of the first implementations of reduced representation, high‐throughput sequencing methods using museum specimens (Linck et al., 2017; McCormack et al., 2016), and some of the largest studies implementing WGS of hDNA to date (Irestedt et al., 2022). In this study, we aim to maximize the potential of hDNA from bird study skins by identifying whether DNA source and library preparation method influence the endogenous DNA content and sequencing efficiency of hDNA libraries, and by introducing a predigestion step prior to DNA extraction to reduce exogenous DNA.

In this study, we test three library preparation methods that vary in (1) the number of cleanups and tube transfers that occur before the library amplification step (one vs. two) and (2) whether they convert single‐ or double‐stranded DNA into library molecules. Each cleanup and tube transfer is an opportunity to lose DNA molecules of the target length (greater than number of sequencing cycles) due to the inherent imprecision of SPRI bead cleanups in addition to pipette error. Methods that are optimized with one, in comparison to two, tube transfers should transform more DNA molecules of target length into library molecules, thus maximizing library complexity and sequencing efficiency. Double‐stranded DNA library preparation methods cannot convert ssDNA into library molecules, though as described above, hDNA is expected to consist in some proportion of single‐strand molecules due to degradation. Methods that convert both ssDNA and dsDNA molecules into library should increase the total number of input hDNA molecules that are converted into library. Thus, we expect that dsDNA libraries prepared from hDNA will have reduced sequencing efficiency and possibly endogenous DNA content compared to that of ssDNA libraries.

**FIGURE 1:**
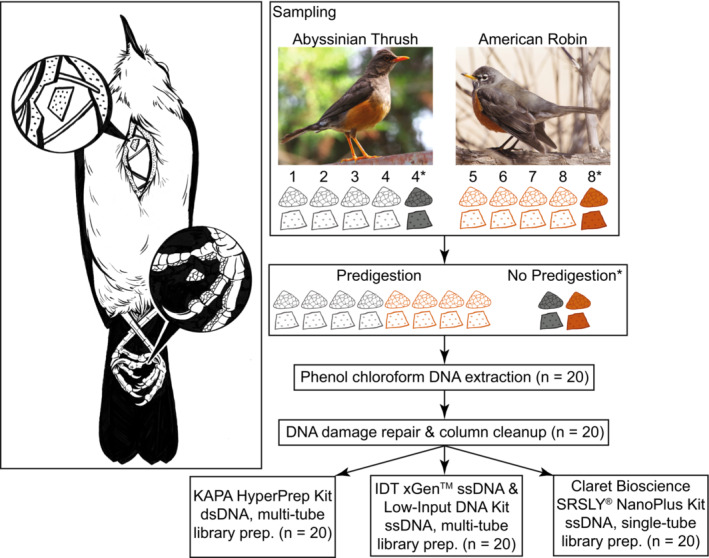
Study sampling design. The illustration on the left shows where incision‐line skin clips and toepads are sampled from the specimens. The flowchart on the right is an overview of the experimental design and the laboratory procedures. Toepads and skin clips are depicted as triangular and trapezoidal shapes, respectively. Photographs by JDM and illustrations by L. Nassef.

We also test the influence of DNA source—toepads versus incision‐line skin clips—on endogenous DNA content and sequencing efficiency. A previous study indicated that toepads consist of longer DNA fragments than skin clips (Tsai et al., 2020), another possible source of hDNA from birds (Töpfer et al., 2011). Libraries prepared from samples consisting of longer DNA fragments should maximize the sequencing capacity, resulting in longer read lengths on average and greater sequencing efficiency. To test these expectations, we prepared shotgun DNA libraries from a toepad and skin clip from eight approximately 100‐year‐old bird specimens via three methods: (1) dsDNA, single tube (KAPA HyperPrep Kit); (2) ssDNA, multi‐tube (IDT xGen™ ssDNA & Low‐Input DNA Library Prep Kit); and (3) ssDNA, single tube (Claret Bioscience SRSLY® NanoPlus Kit). We sought to reduce exogenous DNA by implementing a predigestion step modified from existing aDNA methods, to our knowledge, for the first time on bird specimens. To qualitatively evaluate the influence of the predigestion we also prepared libraries from replicate toepad and skin clip DNA extractions not subjected to predigestion from two of the eight specimens (Figure 1).

## 2 METHODS

### 2.1 Sampling

We sampled eight bird specimens: four Abyssinian Thrush (*Turdus abyssinicus*; hereafter thrushes) and four American Robin (*Turdus migratorius*; hereafter robins; Table 1). We chose the thrush specimens based on their inclusion in another ongoing project and chose to bolster our sample size for this study with the robin specimens because they are a closely related, similar species that is well‐represented in North American natural history collections. Moreover, the thrushes were collected in the tropics and the robins were collected in a temperate region which could influence the drying time of the study skins and in turn, possible degradation due to rot or the microbial load within dried skin (Irestedt et al., 2022). We chose specimens collected within 1 year of each other to control for DNA degradation due to time since specimen preparation.

**TABLE 1:** Summary of extracted DNA and sequencing results for each sample.

Species	Sample	Post‐extraction		Post‐repair		Method	Type	Raw reads (millions)	Clean reads (millions)	Mapped reads (millions)	Mean read length	Dist. to ref.	Endog. DNA content	Seq. Eff.
		Yield (ng/mg)	Mean DNA size (bp)	Yield (ng/mg)	Mean DNA size (bp)									
*Turdus abyssinicus*	83107	400.03	57.36	136.10	68.02	IDT	Skin	10.54	3.38	2.96	71.99	0.018	0.851	0.193
*T. abyssinicus*	83107					KAPA	Skin	60.29	15.17	12.45	59.75	0.014	0.820	0.119
*T. abyssinicus*	83107					SRSLY	Skin	12.85	5.23	4.64	75.14	0.015	0.822	0.267
*T. abyssinicus*	83107	698.97	65.75	332.89	83.90	IDT	Toepad	12.62	5.51	5.12	97.84	0.017	0.900	0.382
*T. abyssinicus*	83107					KAPA	Toepad	85.06	28.05	25.05	78.48	0.012	0.889	0.224
*T. abyssinicus*	83107					SRSLY	Toepad	8.81	3.36	2.89	67.02	0.017	0.853	0.213
*T. abyssinicus*	83109	376.78	57.93	73.28	61.74	IDT	Skin	11.26	3.29	1.69	62.71	0.035	0.480	0.086
*T. abyssinicus*	83109					KAPA	Skin	50.69	12.09	5.77	53.49	0.027	0.477	0.057
*T. abyssinicus*	83109					SRSLY	Skin	10.08	3.74	2.13	65.56	0.030	0.519	0.119
*T. abyssinicus*	83109	858.85	66.68	338.13	85.08	IDT	Toepad	10.95	4.56	4.18	88.02	0.018	0.896	0.323
*T. abyssinicus*	83109					KAPA	Toepad	63.05	21.06	18.86	79.73	0.013	0.890	0.232
*T. abyssinicus*	83109					SRSLY	Toepad	9.29	3.46	2.97	66.34	0.017	0.853	0.206
*T. abyssinicus*	83114	1350.36	78.71	576.64	218.79	IDT	Skin	46.55	32.78	30.90	100.17	0.009	0.916	0.640
*T. abyssinicus*	83114					KAPA	Skin	32.03	15.21	14.27	109.38	0.013	0.919	0.472
*T. abyssinicus*	83114					SRSLY	Skin	9.56	5.06	4.69	104.24	0.017	0.903	0.503
*T. abyssinicus*	83114	800.00	53.96	55.17	98.74	IDT	Toepad	24.48	10.02	8.80	89.44	0.016	0.856	0.309
*T. abyssinicus*	83114					KAPA	Toepad	45.38	15.44	13.93	83.94	0.015	0.889	0.250
*T. abyssinicus*	83114					SRSLY	Toepad	7.62	3.34	3.01	85.32	0.018	0.874	0.332
*T. abyssinicus*	83114^a^	4254.55	96.90	1872.73	211.98	IDT	Skin	42.34	28.30	26.73	101.24	0.009	0.917	0.615
*T. abyssinicus*	83114^a^					KAPA	Skin	34.26	15.74	14.75	107.05	0.013	0.919	0.447
*T. abyssinicus*	83114^a^					SRSLY	Skin	9.35	4.80	4.44	101.67	0.017	0.902	0.474
*T. abyssinicus*	83114^a^	1901.96	56.07	202.94	107.53	IDT	Toepad	28.09	11.70	10.77	99.56	0.015	0.891	0.368
*T. abyssinicus*	83114^a^					KAPA	Toepad	62.51	22.23	20.22	84.52	0.013	0.899	0.266
*T. abyssinicus*	83114^a^					SRSLY	Toepad	14.23	6.31	5.75	85.21	0.016	0.876	0.344
*T. abyssinicus*	83115	942.98	56.71	526.94	69.01	IDT	Skin	14.52	4.28	3.80	78.74	0.017	0.858	0.197
*T. abyssinicus*	83115					KAPA	Skin	80.02	19.29	16.20	59.36	0.014	0.843	0.117
*T. abyssinicus*	83115					SRSLY	Skin	11.55	3.30	2.65	54.80	0.015	0.803	0.120
*T. abyssinicus*	83115	564.00	59.43	217.47	83.13	IDT	Toepad	43.02	17.60	16.22	94.73	0.013	0.893	0.345
*T. abyssinicus*	83115					KAPA	Toepad	14.25	4.92	4.37	78.08	0.018	0.881	0.232
*T. abyssinicus*	83115					SRSLY	Toepad	12.70	5.55	5.02	81.83	0.015	0.862	0.324
*T. migratorius*	162188	375.19	52.58	597.41	58.71	IDT	Skin	15.77	4.77	3.97	60.97	0.018	0.822	0.146
*T. migratorius*	162188					KAPA	Skin	2.14	0.74	0.60	60.52	0.018	0.803	0.162
*T. migratorius*	162188					SRSLY	Skin	9.77	3.52	2.94	61.09	0.018	0.823	0.176
*T. migratorius*	162188	501.80	57.60	436.09	76.70	IDT	Toepad	21.99	9.50	8.58	95.19	0.016	0.879	0.354
*T. migratorius*	162188					KAPA	Toepad	10.85	4.53	3.96	78.38	0.019	0.866	0.275
*T. migratorius*	162188					SRSLY	Toepad	8.36	3.60	3.15	76.19	0.019	0.868	0.279
*T. migratorius*	162188^a^	173.57	54.16	47.37	62.90	IDT	Skin	6.56	4.11	0.86	80.05	0.020	0.174	0.100
*T. migratorius*	162188^a^					KAPA	Skin	14.98	6.38	2.90	59.05	0.018	0.334	0.110
*T. migratorius*	162188^a^					SRSLY	Skin	8.85	3.79	2.07	63.26	0.017	0.415	0.135
*T. migratorius*	162188^a^	231.03	59.13	98.59	77.89	IDT	Toepad	9.14	3.95	3.55	89.65	0.019	0.875	0.333
*T. migratorius*	162188^a^					KAPA	Toepad	31.17	12.31	10.86	78.33	0.017	0.873	0.263
*T. migratorius*	162188^a^					SRSLY	Toepad	10.20	4.42	3.92	78.11	0.019	0.870	0.293
*T. migratorius*	175413	197.25	50.97	59.33	62.37	IDT	Skin	8.18	2.72	2.31	78.30	0.019	0.807	0.208
*T. migratorius*	175413					KAPA	Skin	15.67	4.99	3.86	60.91	0.018	0.770	0.144
*T. migratorius*	175413					SRSLY	Skin	11.30	4.57	3.84	70.22	0.017	0.792	0.227
*T. migratorius*	175413	501.09	56.22	219.23	86.23	IDT	Toepad	12.34	6.03	5.66	100.66	0.017	0.894	0.442
*T. migratorius*	175413					KAPA	Toepad	32.87	13.07	11.71	81.68	0.016	0.882	0.280
*T. migratorius*	175413					SRSLY	Toepad	7.51	3.53	3.24	86.20	0.017	0.868	0.372
*T. migratorius*	175421	463.63	52.32	111.02	62.95	IDT	Skin	6.68	2.31	1.97	64.10	0.019	0.840	0.180
*T. migratorius*	175421					KAPA	Skin	56.18	17.57	14.70	63.39	0.015	0.833	0.159
*T. migratorius*	175421					SRSLY	Skin	14.82	5.83	5.05	67.82	0.017	0.838	0.222
*T. migratorius*	175421	838.10	60.18	234.00	82.44	IDT	Toepad	21.44	9.64	8.98	98.88	0.016	0.891	0.398
*T. migratorius*	175421					KAPA	Toepad	51.02	19.65	17.51	78.30	0.015	0.879	0.259
*T. migratorius*	175421					SRSLY	Toepad	7.29	3.33	3.03	83.03	0.018	0.868	0.343
*T. migratorius*	66823	1150.86	387.24	950.45	1923.43	IDT	Skin	60.98	51.78	49.16	95.45	0.007	0.920	0.736
*T. migratorius*	66823					KAPA	Skin	35.56	24.12	22.91	106.55	0.010	0.921	0.659
*T. migratorius*	66823					SRSLY	Skin	9.88	6.23	5.87	105.58	0.017	0.917	0.605
*T. migratorius*	66823	223.70	53.26	47.36	75.28	IDT	Toepad	5.13	2.24	2.03	85.15	0.020	0.885	0.321
*T. migratorius*	66823					KAPA	Toepad	50.77	19.75	17.26	73.40	0.015	0.871	0.241
*T. migratorius*	66823					SRSLY	Toepad	11.86	4.31	3.63	61.16	0.018	0.855	0.182

^a^ Extraction replicate not subjected to predigestion.

We collected two samples from each specimen—a toepad and a skin clip from the incision‐line through the pectoral apterium (following Tsai et al., 2020)—to evaluate whether tissue source differed in proportion of endogenous DNA and sequencing efficiency. Toepads and skin clips were approximately 3 × 3 mm (mean, *M* = 2.25 mg; standard deviation, SD = 1.01) and 5 × 5 mm (*M* = 2.33, SD = 1.91), respectively, though exact sizes varied so we weighed each sample to standardize our measures of DNA yield below (Table S1). We also took replicate samples from one specimen of each species (Table 1) to qualitatively evaluate the effect of sample predigestion prior to DNA extraction on the exogenous DNA load. The experimental design is summarized in Figure 1.

We followed stringent sampling precautions to limit the introduction of contaminant DNA: We (1) wore surgical masks and gloves throughout sampling, (2) took samples in the collections away from any specimen preparation laboratory, (3) did not enter any modern molecular DNA or specimen preparation laboratory prior to sampling, (4) prepared the work surface and all other supplies (e.g., forceps, optivisor, writing utensil) by cleaning with freshly prepared 10% bleach followed by 70% isopropanol or ethanol, and (5) immediately placed samples in sterile microcentrifuge tubes that were unpackaged in a sterile laboratory and not opened prior to beginning molecular laboratory work. We used a fresh pair of gloves and sterile scalpel blade for each sample to minimize contamination between samples.

### 2.2 Molecular laboratory work

We followed stringent ancient DNA clean laboratory protocols to minimize contamination during molecular laboratory work (Fulton & Shapiro, 2019). We completed all pre‐PCR steps in an ancient DNA facility in the Department of Human Genetics at the University of Chicago in a non‐human specific room. We performed each step prior to PCR in a maximum batch size of 12 samples, introduced a negative control in each batch of extractions and library preparations, and then carried these controls through the remaining steps of laboratory work.

Prior to DNA extraction, we wanted to apply a pretreatment to remove exogenous DNA from the sample surface. Several pretreatments to reduce exogenous DNA from teeth and bone powder have been developed in the fields of forensics and aDNA, including but not limited to: (1) scraping the surface from the sample, (2) wiping the sample surface with dilute bleach, (3) mixing the sample in its entirety in a diluted bleach or phosphate buffer for fifteen minutes followed by rinsing (Korlević et al., 2015), (4) enzymatic predigestion in the digestion buffer for a reduced time and temperature (Damgaard et al., 2015), and (5) a combination of the previous methods (e.g. Boessenkool et al., 2016). We were cautious in applying one of these treatments to toepads and skin clips for the first time because these samples are dried skin and flesh and thus more delicate compared to the powdered bone, bone fragments, or teeth that these pretreatments are typically applied to by aDNA researchers. Also, the starting material for each toepad and skin clip is much smaller than that of a typical sample taken from bone. Given these considerations, we chose a predigestion rather than a more aggressive chemical or mechanical pretreatment. Previous research on predigestion of bone powder and teeth has shown an increase in endogenous DNA content after as little as 15–30 min of predigestion and that longer predigestion times can reduce endogenous DNA content and drive down library complexity (Damgaard et al., 2015). The standard protocol suggests considering input amount and preservation when choosing a predigestion time between 15 and 30 min (Schroeder et al., 2019). Again, given the delicate nature and smaller size of our samples, we predigested each sample for up to 15 min in 180 μL of digestion buffer and 12 mAU proteinase K at 37°C while rotating at 11 *g*. To prevent significant digestion of the sample, we checked each predigesting sample regularly and removed samples beginning after the first minute. We applied this predigestion treatment to all but the four replicate samples described above (Figure 1).

We extracted DNA from all samples via a phenol‐chloroform protocol followed by ethanol precipitation as in Tsai et al. (2020) with minor modifications: (1) we used a digestion buffer prepared in house and comprising 30 mM Tris–HCl, 10 mM EDTA, 1% SDS instead of the Qiagen Buffer ATL, (2) we began samples digestion with 40 μL rather than 20 μL of proteinase K, (3) we did not include DTT in our digestion solution, and (4) we did not mash tissue during digestion. We performed an NEB PreCR DNA repair treatment following the sequential reaction protocol on each DNA extraction. This treatment repairs DNA damage from hydrolysis and oxidative stress, among other mechanisms, that result in deaminated cytosines, nicks, and other DNA damage incurred with age. A previous study found that a different NEB repair kit optimized for formalin‐fixed specimens increased the yields of libraries prepared from historical beetle specimens by approximately 30% (Sproul & Maddison, 2017). Following the damage repair treatment, we performed a Qiagen MinElute column cleanup and resuspended the DNA in 50 μL of PCR‐grade water. Next, we measured the DNA yield and distribution of DNA fragment sizes using the Qubit High Sensitivity dsDNA (Thermo Fisher Scientific) and Agilent Bioanalyzer High Sensitivity DNA Kit assays, respectively, following the DNA extraction and again following DNA repair and cleanup. We performed the same assays for each extraction negative control to monitor for contamination.

We prepared three shotgun sequencing libraries from each DNA extraction and negative control. Each of the three libraries was prepared via a different method: (1) dsDNA, single tube (KAPA HyperPrep Kit); (2) ssDNA, multi‐tube (IDT xGen™ ssDNA & Low‐Input DNA Library Prep Kit); and (3) ssDNA, single tube (Claret Bioscience SRSLY® NanoPlus Kit). We largely followed manufacturer protocols with the following modifications: during the KAPA adapter ligation step, we ligated 25 μM iTru Stubs (Glenn et al., 2019) to each library molecule. For all cleanups we used a homebrew SPRI bead solution (Rohland & Reich, 2012), and for each cleanup step in the KAPA and IDT protocols we performed 1.2× SPRI concentration cleanups. We indexed each library via amplification with 2.5 μM of a unique pair of iTru5 and iTru7 indexed primers (Glenn et al., 2019) and KAPA HiFi HotStart Uracil + ReadyMix. For amplification we split each adapter‐ligated library into two replicates of 25 μL each and ran a nine‐ to twelve‐cycle PCR, depending on input DNA amount and method, on the first replicate; then we estimated the yield of the first replicate via a Qubit High Sensitivity dsDNA assay and ran a 10‐ to 12‐cycle PCR on the second adapter‐ligated library replicate. We combined amplified replicates for each library and performed a final SPRI cleanup. Finally, we measured the average molecule size and calculated the concentration of adapter‐ligated molecules for each sample library via an Agilent Bioanalyzer High Sensitivity DNA Kit assay and qPCR with the KAPA Library Quantification Kit. We submitted a final library pool to Texas Tech University Center for Biotechnology & Genomics for sequencing. They first checked that libraries were sequencable with an Illumina MiSeq nano run followed by 100 base pair (bp) paired‐end sequencing on one Illumina NovaSeq SP flowcell.

### 2.3 Bioinformatics

We received demultiplexed sequence data as raw fastq files from the sequencing facility and ran FastQC (https://www.bioinformatics.babraham.ac.uk/projects/fastqc/) to assess quality and adapter contamination by library preparation method. We trimmed 10 bp from the beginning of every IDT library read 2 via Seqtk *trimfq* (https://github.com/lh3/seqtk) to remove a low‐complexity polynucleotide tail that facilitates adapter ligation in this method. Duplicate reads resulting from PCR were identified and removed via Super Deduper (https://github.com/s4hts/HTStream). Then we used SeqPrep (https://github.com/jstjohn/SeqPrep) to simultaneously identify adapter contamination and overlapping paired reads, and then trim adapters and merge reads as necessary. We trimmed bases from both read ends via 4 bp sliding window to a minimum quality of 15 and then removed reads that were less than 30 bp long via Trimmomatic v0.39 (Bolger et al., 2014). Finally, we removed any remaining reads that were comprised of more than 50% of one nucleotide via remove_low_complex.py (distributed as part of the NF‐Polish sequence polishing pipeline described in Irestedt et al. (2022)). We aligned cleaned sequencing reads to the Rufous‐bellied Thrush (*T. rufiventris*) reference genome (ASM1318643v1) via BWA 0.7.17 *mem* (Li, 2013) and indexed mapped reads with Samtools 1.9 *index* (Danecek et al., 2021; Li et al., 2009). Following sequence cleaning and alignment we evaluated adapter contamination and sequence quality via FastQC. We used MapDamage 2.0 (Ginolhac et al., 2011; Jónsson et al., 2013) to estimate the frequency of C to T and G to A misincorporations that result from a transition to uracil via hydrolysis during DNA degradation over time. For each library, we output sequencing metrics via Samtools 1.9 *stats* and estimated sequencing efficiency and endogenous DNA content as the proportion of raw and cleaned bases, respectively, that aligned to the reference genome. This measure of endogenous DNA content is likely an underestimate of the true proportion of endogenous DNA in our samples because some endogenous DNA sequences are lost during bioinformatics processing due to the minimal sequence length that can be mapped with a given algorithm and the genetic distance of the sample from the reference genome.

### 2.4 Analyses

We tested whether there were differences in DNA yield and mean DNA fragment size between different sources (toepad vs. skin clip) via paired *t*‐tests. We evaluated whether DNA source, library preparation method, or an interaction between them resulted in differences in (1) endogenous DNA content, (2) sequencing efficiency, and (3) mean read length via two‐way, repeated measures ANOVAs. For any two‐way ANOVA that resulted in a significant interaction, we performed a one‐way, repeated measures ANOVA for each method to evaluate whether there were significant differences by DNA source. For any two‐way ANOVA that resulted in a significant effect of either independent variable, we performed subsequent pairwise, paired *t*‐tests between all library preparation methods. We accounted for multiple testing in all post‐hoc one‐way and paired *t*‐tests by adjusting *p*‐values via the BH method (Benjamini & Hochberg, 1995).

We expected that samples with larger mean DNA fragment sizes would also have longer mean read lengths and as a result, greater endogenous DNA content and sequencing efficiency. To test this hypothesis while controlling for any effect of DNA source and library preparation method we defined two linear models for each of the following response variables: endogenous DNA content, sequencing efficiency, and read length. Each null model included the library preparation method and DNA source as fixed effects and sample as a random effect. The alternative model also included mean DNA fragment length as a fixed effect. To test whether mean DNA fragment length had a significant influence on each response variable we performed a likelihood ratio test of the null and alternative model.

Finally, we sought to qualitatively evaluate the effect of predigestion on replicate samples. To do so, we plotted the difference in each metric of interest between the replicate samples. All statistical analyses were completed in R v4.1.0 (R Core Team, 2021). ANOVAs and *t*‐tests were conducted with the package *rstatix v0.7.0* (Kassambara, 2021), linear mixed models were built in *lme4* v1.1–27.1 (Bates et al., 2015), and we used the suite of functions in *tidyverse* v1.3.1 (Wickham et al., 2019) for data parsing, manipulation, and visualization.

## 3 RESULTS

### 3.1 DNA yield and size

**FIGURE 2:**
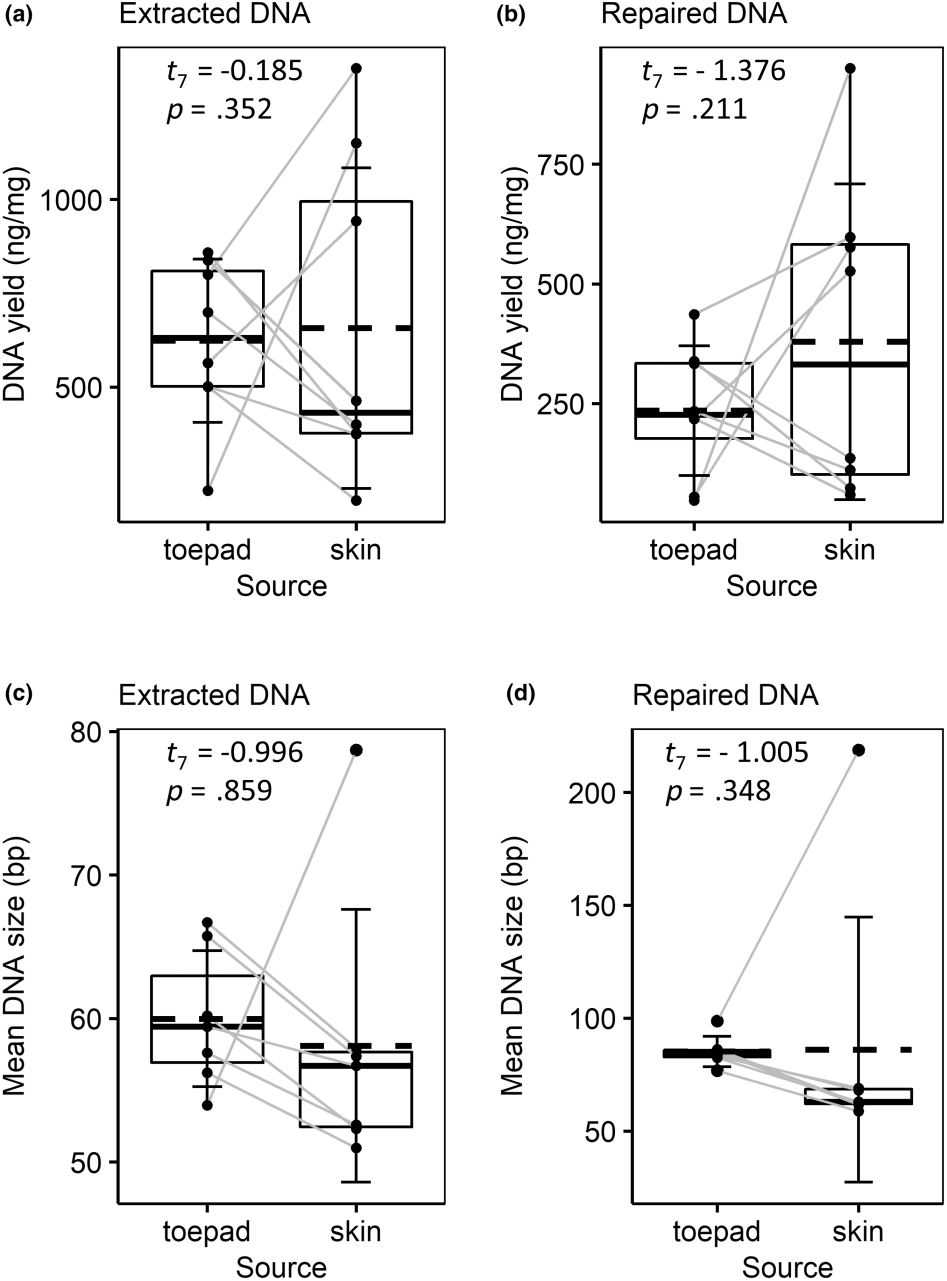
Source tissue impacts on DNA quantity and quality. For each sample, DNA yield (ng/mg) measured (a) following DNA extraction and (b) following DNA repair and cleanup is plotted by DNA source (toepad or skin clip). For each sample, mean DNA fragment size (bp) (c) following DNA extraction and (d) following DNA repair are plotted by DNA source. Samples from robin specimen 66823 are not plotted in (c) and (d) because the mean DNA size of its skin clip (Table 1) limits visualization of the variation in the size of the other samples. Gray lines connect toepad and skin clip data points from the same specimen. Summary statistics are also plotted by source: the mean and median are represented by dashed and solid lines, respectively, the upper and lower limits of the boxes represent the 75% and 25% quantiles, and the error bars represent the standard deviation.

All 60 DNA extractions were successful in terms of producing measurable amounts of DNA with an average of 589.1 nanograms (ng) per sample and a minimum of 48.6 ng in the skin clip control replicate from robin specimen 162188 (Table 1). All samples retained enough DNA through the DNA repair and cleanup to progress to library preparation by each of the three methods. In general, the DNA repair resulted in an upshift in the distribution of DNA fragment lengths (Figures S1A and S2A). There was no statistical difference in DNA yield and size between toepad and skin clips immediately following extraction or after the DNA repair and cleanup (Figure 2). However, the lack of statistical difference in DNA size is driven by the large variance in skin clips (Table 2). The mean size of DNA extracted from the toepad sample is greater than that of the skin clip sample for all but two specimens: robin specimen 83114 and thrush specimen 66823 (Table 1). For example, these two specimens bias the distribution of the post‐repair skin clip mean size (post‐repair *M* = 315.63; median, Mdn = 65.49; SD = 651.92) upwards, but not the toepad mean size (post‐repair *M* = 83.94, Mdn = 83.94, SD = 7.13).

**TABLE 2:** Summary (median, mean, and standard deviation) of DNA yield and mean DNA size for predigested toepads and skin clips.

Source	Yield (ng/mg)							
	Post‐extraction		Post‐repair		Post‐extraction		Post‐repair	
	Mdn	*M* (SD)	Mdn	*M* (SD)	Mdn	*M* (SD)	Mdn	*M* (SD)
Toepad (*n* = 8)	631.49	623.314 (217.606)	226.62	235.042 (135.626)	58.52	59.136 (4.985)	83.51	83.937 (7.132)
Skin (*n* = 8)	431.83	657.135 (427.479)	331.52	378.896 (329.864)	57.04	99.227 (116.707)	65.49	315.628 (651.918)

### 3.2 Endogenous DNA content and sequencing efficiency

Sequencing returned a total of approximately 1.45 billion raw reads and, per library, an average of 11.23 million raw reads (SD = 1.84) and 9.88 million mapped reads (SD = 9.78) per library excluding non‐predigested replicates. Detailed sequencing metrics for each library are reported in the supplementary material (Table S1).

**FIGURE 3:**
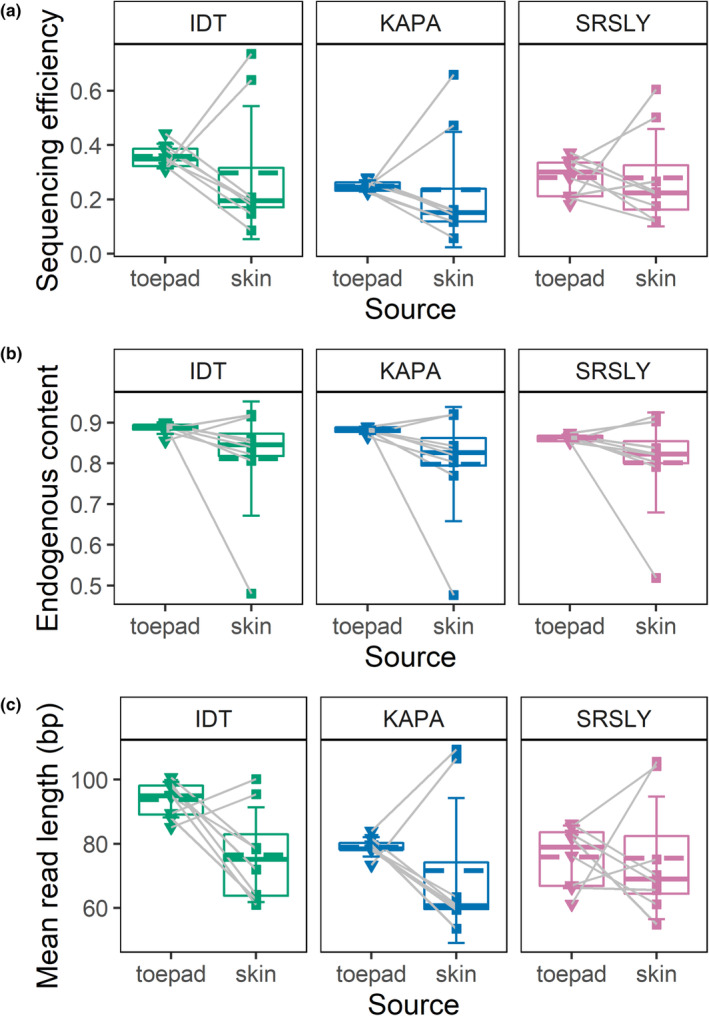
Association of sequencing library type and sequence data characteristics. For each library, (a) sequencing efficiency, or the proportion of raw bases that uniquely mapped to the reference genome, (b), endogenous DNA content, or the proportion of cleaned bases that uniquely mapped to the reference genome, and (C) the mean read length (bp) is plotted by DNA source (toepad or skin clip). Gray lines connect toepad and skin clip data points from the same specimen. Summary statistics are also plotted by method and source: the mean and median are represented by dashed and solid lines, respectively, the upper and lower limits of the boxes represent the 75% and 25% quantiles, and the error bars represent the standard deviation.

There was a significant difference across library preparation methods, but not DNA source in endogenous DNA content and sequencing efficiency with IDT outperforming SRSLY and KAPA in both metrics (Table 3). Similar to the results described above for DNA size, the toepad samples outperform the corresponding skin clip samples except for specimens 83114 and 66823 (Figure 3a,b) so we summarize the results by method and source (Table 4). The average endogenous DNA content of IDT toepad and skin clip libraries is 88.7% (SD = 0.014) and 81.2% (SD = 0.140) respectively, 0.06% and 1.4% greater than that of KAPA, and 2.4% and 1% greater than that of SRSLY. The average sequencing efficiency of IDT toepad and skin clip libraries is 35.9% (SD = 0.045) and 29.8% (SD = 0.245), respectively. In comparison to IDT, KAPA toepad and skin clip libraries are 11% and 1.8% less efficient, respectively, and SRSLY toepad and skin clip libraries are 7.8% and 1.8% less efficient. There was also a significant difference among methods in mean read length with IDT producing longer reads than KAPA and SRSLY with a significant interaction between method and DNA source (Tables 3 and 4, Figure 3c). IDT toepad libraries produced significantly longer reads than IDT skin clip libraries (Tables 3 and 4, Figure 3c).

**TABLE 3:** Summary of the statistical analyses of endogenous DNA content, sequencing efficiency, and mean read length and their results. All the *p*‐values presented for *t*‐tests are corrected for multiple testing via the Benjamini and Hochberg (1995) method.

Response variable	Test	df	Test‐statistic	*p*‐value
Endogenous DNA content	ANOVA–source	1,7	0.066	.805
	ANOVA–method	2,14	6.241	.012
	ANOVA–source:method	2,14	0.739	.496
	*T*‐test–IDT versus KAPA	15	6.631	<.001
	*T*‐test–IDT versus SRSLY	15	2.476	.026
	*T*‐test–KAPA versus SRSLY	15	−2.570	.026
Sequencing efficiency	ANOVA–source	1,7	0.066	.805
	ANOVA–method	2,14	6.241	.012
	ANOVA–source:method	2,14	0.739	.496
	*T*‐test–IDT versus KAPA	15	2.492	.037
	*T*‐test–IDT versus SRSLY	15	2.765	.037
	*T*‐test–KAPA versus SRSLY	15	1.283	.219
Mean read length (bp)	ANOVA–source	1,7	1.333	.286
	ANOVA–method	2,14	14.181	<.001
	ANOVA–source:method	2,14	6.778	.009
	*T*‐test–IDT versus KAPA	15	3.879	.004
	*T*‐test–IDT versus SRSLY	15	3.001	.013
	*T*‐test–KAPA versus SRSLY	15	−0.184	.856
Mean read length (bp)—IDT	ANOVA–source	1,7	7.179	.096
Mean read length (bp)—KAPA	ANOVA–source	1,7	0.818	1.0000
Mean read length (bp)—SRSLY	ANOVA–source	1,7	0.002	1.0000

**TABLE 4:** Summary (median, mean, and standard deviation) of sequencing metrics for predigested toepads and skin clips.

Source	Mean read length (bp)						Endogenous DNA content						Sequencing efficiency					
	IDT		KAPA		SRSLY		IDT		KAPA		SRSLY		IDT		KAPA		SRSLY	
	Mdn	M (SD)	Mdn	M (SD)	Mdn	M (SD)	Mdn	M (SD)	Mdn	M (SD)	Mdn	M (SD)	Mdn	M (SD)	Mdn	M (SD)	Mdn	M (SD)
Toepad (*n* = 8)	94.959	93.74 (5.598)	78.428	78.998 (3.059)	79.005	75.883 (9.771)	0.892	0.887 (0.014)	0.882	0.881 (0.009)	0.865	0.863 (0.008)	0.35	0.359 (0.045)	0.245	0.249 (0.021)	0.302	0.281 (0.072)
Skin (*n* = 8)	75.145	76.554 (14.799)	60.717	71.669 (22.587)	69.018	75.557 (19.097)	0.846	0.812 (0.14)	0.827	0.798 (0.14)	0.823	0.802 (0.123)	0.195	0.298 (0.245)	0.151	0.236 (0.212)	0.225	0.28 (0.179)

The tests of the influence of input DNA fragment size on sequencing outcomes produced mixed results. Including DNA size significantly improved the fit of the linear models for endogenous DNA content ($\chi_{1}^{2}$ = 7.558, *p* = .006), sequencing efficiency ($\chi_{1}^{2}$ = 45.771, *p* < .001), and read length ($\chi_{1}^{2}$ = 25.308, *p* < .001). However, in the linear models including DNA size, it was only a significant predictor of endogenous DNA content and read length based on confidence intervals of the coefficient estimate. One to two toepad samples drive the relationship between DNA size and sequencing efficiency to be negative for the IDT and KAPA methods and also drive the relationship between DNA size and mean read length to be negative for IDT (Figure S3).

Two of the 20 libraries exhibited low endogenous DNA content compared to all others. The skin clip library of thrush specimen 83109 exhibited approximately 1% more differences from the reference genome than all other samples (Table 1, Figure S4) in addition to a comparatively low proportion of endogenous DNA content across preparation methods (IDT = 48.0%, KAPA = 47.7%, SRSLY = 51.9%). The replicate skin clip library of robin specimen 162188 that was not subjected to predigestion also had a low proportion of endogenous DNA content across preparation methods (IDT = 17.4%, KAPA = 33.4%, SRSLY = 41.5%), but it was similar to all other libraries in its genetic distance from the reference (Figure S4). The high‐performing skin clips samples from specimens 83114 and 66823 that biased the skin clip averages of most metrics upward also had similar genetic distances to the reference (Table 1, Figure S4).

The influence of sample predigestion was unclear (Table 1, Figure S5). The differences in DNA yield, mean DNA size, and endogenous DNA content were inconsistent between the control and predigested replicates from robin 162188 and thrush 83114. The difference between the replicates in sequencing efficiency and mean read length were marginal.

## 4 DISCUSSION

### 4.1 WGS of hDNA from 100‐year‐old bird study skins

We have demonstrated via shallow sequencing of 60 hDNA libraries that ssDNA library preparation methods outperform dsDNA methods in sequencing efficiency and, to a lesser extent, in returning endogenous DNA content from WGS ~100‐year‐old bird specimens. In contrast to our predictions, the IDT multi‐tube, ssDNA method outperformed the Claret Biosciences single‐tube, ssDNA method and we discuss possible explanations below. We also confirm previous research that suggested that toepads provide consistently larger DNA fragments (Tsai et al., 2020) and demonstrate that hDNA from toepads rather than skin clips is a better source for WGS. We show that, though skin clips may sometimes outperform toepads for a given specimen, toepads have less variance and therefore less unexpected sequencing outcomes. Altogether, we have shown that toepads are a better source of DNA, and ssDNA library preparations are a better method for collecting WGS from 100‐year‐old bird specimens. Below we elaborate further on the nuances of our findings and conclude with broader implications for WGS of historical DNA from museum specimens in natural history collections.

### 4.2 Library preparation method and DNA source influenced sequencing

Library preparation method influenced all key metrics; but contrary to our predictions, the SRSLY ssDNA, single‐tube method did not outperform the other two methods. Instead, the IDT ssDNA, multi‐tube method resulted in greater endogenous DNA content, sequencing efficiency, and read lengths than either of the other methods (Figure 3, Table 3). The margin of difference between IDT and the other methods was much greater for sequencing efficiency and average read length than endogenous DNA content (Table 4), suggesting that IDT produced more complex libraries. The only other study to compare ssDNA and dsDNA methods for shotgun sequencing of historical specimens prepared libraries from beetle specimens of various ages using the same ssDNA, multi‐tube method that we implemented and a different dsDNA, single‐tube method (Sproul & Maddison, 2017). Those results showed no difference in endogenous DNA content between methods, and that ssDNA libraries maintained more sequencing reads through quality filtering and trimming. We find this consistent with our results and suspect that controlling for taxonomy and specimen age in our experimental design facilitated detecting the small difference that library preparation method made in endogenous DNA content. That IDT outperformed SRSLY makes some sense given that SRSLY was originally developed for cell‐free DNA which averages 30 bp long (Troll et al., 2019); though the commercial kit provides several versions of the protocol optimized for different purposes, and we implemented the version for moderate‐length DNA inserts, less than 200 bp. That protocol for moderate‐length DNA inserts includes two‐sided SPRI cleanups following adapter ligation and indexing PCR, as compared to the IDT ssDNA method which uses single‐sided cleanups. We suspect that the two‐sided cleanups in SRSLY limited conversion of DNA molecules on the larger end of the DNA size distribution into library molecules (Figures S1 and S2). This is consistent with SRSLY producing shorter average read lengths than IDT. Still, SRSLY outperformed the KAPA dsDNA method overall in sequencing efficiency and outperformed IDT in sequencing efficiency for skin clips in a few specimens (Figure 3a). It is possible that SRSLY may be the better method for samples that are more degraded as a result of age or DNA source. More recently another ssDNA single‐tube method that builds upon SRSLY was developed specifically for ancient DNA samples (Kapp et al., 2021); though the nonproprietary status limits ease of implementation. It may be worthwhile to optimize the SRSLY cleanup steps to maximize conversion of the largest DNA fragments as the IDT method costs 1.89× more than SRSLY per reaction.

Most statistical tests of the effect of DNA source on our metrics of interest were not significant, though toepads seem to perform better for WGS when considering the influence of the large variance in the skin clip metrics. Toepads had much smaller variance in DNA yield, DNA size, endogenous DNA content, sequencing efficiency, and average read length than skin clips. For six of the eight specimens, toepads clearly provided greater endogenous DNA content, sequencing efficiency, and average read length than the corresponding skin clip. In general, this result is reflected by the mean, and more so the median values, of these metrics for toepads as compared to skin clips (Tables 2 and 4). That phenol‐chloroform extraction of toepads does not yield more DNA but does result in longer DNA fragments than that of skin clips supports previous research (Tsai et al., 2020). Our results are also in line with previous studies based on five fluid‐preserved garter snake specimens (Zacho et al., 2021) and three dried mammal skins from different species (McDonough et al., 2018) that showed that different DNA sources have differences in endogenous DNA content. It is possible that hDNA sampled from bird specimen toepads produces better WGS data than that of skin clips because of their different structural makeups. The keratinized, scaly skin of bird feet may provide a better environmental barrier to water—which promotes DNA degradation via hydrolysis and also overall tissue degradation—and to invading microbes that would increase exogenous DNA. Toepad cells may also have lower innate water content due to desiccation during keratinization (Bengtsson et al., 2012). The role of the keratin structure in maintaining better DNA for WGS is supported by the work of McDonough et al. (2018) which showed that of bone, skin, and claw samples from dried mammal specimens, claw samples had the highest or near highest proportion of endogenous DNA and in a qPCR analysis, the highest copy number of nuclear genomic markers.

### 4.3 Predigestion, high‐performing samples, and potential contamination

Interpretation of the effect of predigestion on reducing exogenous DNA content and increasing sequencing efficiency is limited by the small sample size for which we sequenced replicate predigested and control samples. The lack of clear signal of predigestion effect in DNA yield and size is unsurprising given the variation across all samples (Figure 2). Moreover, the lack of any potential signal is unsurprising given that of the eight samples included as replicates to evaluate predigestion, three were exceptions to the general trends identified by the larger study. Both skin clips from thrush 83114 were among the few skin clip samples that performed uncharacteristically better than all other samples and the control skin clip replicate from robin from 162188 resulted in the lowest endogenous DNA content of all libraries (Table 1) indicating high levels of exogenous DNA or contamination. Notably, the predigested skin clip replicate sample from robin 162188 did not show signs of contamination suggesting that predigestion may have reduced exogenous DNA in only 2 min of predigestion time. In contrast, the skin clip from thrush specimen 83109 was one of four samples with the shortest, one‐minute predigestion and also showed a clearer signal of contamination based on low endogenous DNA content and a larger genetic distance from the reference than other samples (Table 1, Figure S4). Finally, it is likely that predigestion reduced total DNA yield, though enough DNA remained for all 20 samples to prepare three successfully sequenced libraries. Altogether we suggest this preliminary investigation is a promising avenue to maximize endogenous hDNA from museum bird specimens for WGS and warrants further research.

The primary source of the larger variance in most metrics for skin clips were two specimens for which the skin clips not only outperformed the corresponding toepad from the same specimen but all other samples. Robin 66823 and thrush 83114 returned the first and second largest DNA yield, DNA size, endogenous DNA content, average read length, and highest sequencing efficiency. Notably, thrush 83114 was one of the two specimens included in the predigestion study, and both the predigested and non‐predigested skin clips were high‐performing samples and had consistent values across metrics. There are multiple explanations for why the samples outperformed all others, the first being contamination. However, neither of these samples shows clear signs of contamination like the lower endogenous DNA content or large genetic distance from the reference genome mentioned for the skin clip from thrush 83109. The only explanation of contamination we consider plausible is contamination by modern DNA from the same or a closely related species, perhaps from a more recently collected specimen in the same drawer. This possibility highlights the need for stringent sampling procedures during sample collection from museum specimens for hDNA purposes. Importantly, a population genomics study involving deeper sequencing of these samples would allow assembly of mitochondrial genomes that would enable identification of multiple individuals within one sequencing library. Another possible explanation for the higher performance of these skin clips is that these specimens received different treatments at the time of collection than the other specimens in the study. At the time of collection, it was common practice to treat bird (and mammal) skins with arsenic‐containing solutions for tanning as well as protection from pests (Marte et al., 2006), and arsenic has been demonstrated as a DNA polymerase inhibitor (Töpfer et al., 2011). It is possible that the specimens with high‐performing skin clips were accidentally skipped in some treatment that ultimately promoted DNA damage in the other skins. The last and, in our opinion, most likely possibility is that this variation represents real variation in DNA quality between specimens. Such large variations are not uncommon when working with hDNA (e.g. McDonough et al., 2018) and ancient DNA (e.g. Wales et al., 2015) and highlights the value of identifying methods and DNA sources that can consistently return an expected amount of WGS data like we have found for toepads and the IDT ssDNA, single‐tube method.

## 5 CONCLUSION

We have shown that for 100‐year‐old museum bird study skin specimens, of those combinations we tested, the combination of toepads and ssDNA library preparation, in this case the IDT xGen™ ssDNA & Low‐Input DNA Library Prep Kit, provide the best WGS data. Our results regarding toepads, in combination with previous studies of endogenous DNA content in other taxa, can be reasonably extended beyond birds to suggest that keratinous sources of hDNA may be the best source for WGS and should motivate additional investigations of this hypothesis. We also have shown that when comparing WGS from ssDNA and dsDNA methods, ssDNA methods provide a larger increase in sequencing efficiency than endogenous DNA content, suggesting that they successfully convert more hDNA molecules into sequenceable library molecules and likely lead to more complex libraries better suited for WGS at the depth required for population genomic studies. Further study of the impact of library preparation method on sequencing efficiency that controls for variation among specimens and also evaluates the role of age of the specimen is necessary to identify the threshold at which an ssDNA method is or is not warranted. Also, it may be worthwhile to attempt to further optimize SRSLY cleanups to minimize bias against converting larger fragment hDNA molecules into library molecules as SRSLY is currently ~89% less expensive per reaction than the IDT method. Finally, our inclusion of a predigestion step to reduce external exogenous DNA did not yield straightforward results, though it did provide some indication that it limited contamination in one of four samples for which we made a direct comparison. Importantly we showed that a brief (less than 15 min), gentle (37°C incubation) predigestion step does not preclude successful library construction, and thus we will cautiously include this step in our own protocols moving forward. Altogether this study identifies how to maximize WGS data collected from 100‐year‐old bird specimens and provides some general insights on how to increase the quality and quantity of WGS data recovered from hDNA of museum specimens overall.

## AUTHOR CONTRIBUTIONS

**Amie E. Settlecowski:** Conceptualization (equal); data curation (lead); formal analysis (lead); investigation (lead); writing – original draft (lead). **Ben D. Marks:** Conceptualization (equal); funding acquisition (equal); writing – review and editing (supporting). **Joseph D. Manthey:** Conceptualization (equal); formal analysis (supporting); funding acquisition (equal); writing – review and editing (supporting).

## CONFLICT OF INTEREST STATEMENT

IDT has been a corporate sponsor of the Field Museum of Natural History as recently as 2020, but no funds from IDT were used to directly support the research that is the subject of the publication.

## BENEFIT SHARING STATEMENT

We are sharing all research benefits of this project by providing raw data, bioinformatic scripts, and results in public databases. No monetary benefits are expected from these research results.

## Supporting information


Figures S1‐S5

Table S1

## Data Availability

Raw sequence reads are deposited in the NCBI Sequence Read Archive under BioProject PRJNA1002336. Code used in data analysis is available at: github.com/amiesett/WGS‐hDNA‐birds.
